# Litter accumulation alters the abiotic environment and drives community successional changes in two fenced grasslands in Inner Mongolia

**DOI:** 10.1002/ece3.5469

**Published:** 2019-07-23

**Authors:** Dongjie Hou, Weiming He, Changcheng Liu, Xianguo Qiao, Ke Guo

**Affiliations:** ^1^ State Key Laboratory of Vegetation and Environmental Change, Institute of Botany Chinese Academy of Sciences Beijing China; ^2^ University of Chinese Academy of Sciences Beijing China

**Keywords:** bunch grasses, regulating effect, rhizome grasses, soil moisture, soil temperature

## Abstract

Fencing is an effective and practical method for restoring degraded grasslands in northern China. However, little is known about the role of excess litter accumulation due to long‐term fencing in regulating abiotic environment and driving changes in community structure and function. We conducted a three‐year field experiment in two fenced grasslands in Inner Mongolia, and monitored light quantity, soil temperature, and soil moisture continuously, and determined community height, community aboveground net primary productivity (ANPP), and the relative dominance of different plant functional groups. Litter accumulation reduced light quantity and soil temperature but increased soil moisture. The regulating effects of litter accumulation on soil temperature and soil moisture fluctuated temporally and gradually weakened over the growing season. Litter accumulation also altered community vertical structure and function by increasing community height and ANPP. The increase in soil moisture increased the relative dominance of rhizome grasses but suppressed bunch grasses, thereby shifting bunch grass grasslands to rhizome grass grasslands. Our findings provide a potential mechanism for community succession in the context of litter accumulation in fenced grasslands and indicate that the vegetation and ecosystem services of degraded grasslands are improved after appropriate fencing.

## 1 INTRODUCTION

Arid and semiarid grassland ecosystems are one of the main terrestrial ecosystems in China. They account for 40% of the total land area, and about 78% of these grasslands are distributed in northern China (Sun, 2005). Grassland ecosystems provide a series of economic, ecological, and cultural services, such as providing milk and meat for humans, water and soil conservation, and maintaining cultural diversity (Kang, Han, Zhang, & Sun, 2007). However, since the 1970s, intense human activities including overgrazing and farming have resulted in severe grassland degradation in China (Jiang, Han, & Wu, 2006). To restore the vegetation (e.g., community height, cover, and productivity) and soil (e.g., soil bulk density and nutrients) of degraded grasslands, successive governments have focused on grassland restoration. A series of restoration methods have been implemented in northern China, such as fertilization (Perrow & Davy, 2002) and seeding (Barr, Jonas, & Paschke, 2017). However, these restoration methods not only need huge manpower and resources but also have limited applicability. Of all restoration methods, fencing appears to be an effective and practical method for restoring degraded grasslands, especially for those subjected to overgrazing (Jing, Cheng, & Chen, 2013; Wu, Du, Liu, & Thirgood, 2009). However, although long‐term fencing can facilitate the restoration of vegetation and soil in degraded grasslands (Liu, Wu, Su, Gao, & Wu, 2017; Wang et al., 2018), it can also create new issues. For example, long‐term fencing results in excess litter accumulation on the soil surface, particularly in arid and semiarid grasslands.

Plant litter is a key component in arid and semiarid grasslands and indicates the health of these ecosystems (Facelli & Pickett, 1991a; Wang et al., 2011). Litter accumulation has diverse effects on reproduction, interspecific competition, and community structure and function (Lamb, 2008; Ruprecht, Enyedi, Eckstein, & Donath, 2010). For example, litter accumulation usually has negative effects on seedling recruitment because it prevents seeds, especially for large ones, from reaching the soil surface (Hovstad & Ohlson, 2008; Jensen & Gutekunst, 2003; Rotundo & Aguiar, 2005; Ruprecht & Szabó, 2012). It also inhibits seed germination through toxic allelopathy (Bonanomi, Sicurezza, Caporaso, Esposito, & Mazzoleni, 2006; Ruprecht, Józsa, Ölvedi, & Simon, 2010). Litter accumulation can alter species composition (Amatangelo, Dukes, & Field, 2008; Letts, Lamb, Mischkolz, & Romo, 2015; Weltzin et al., 2005), decrease species richness and evenness (Amatangelo et al., 2008; Foster & Gross, 1998), and increase the cover of some species (Weltzin et al., 2005). Community aboveground net primary productivity (ANPP) responds variably to litter accumulation. Some studies report that litter accumulation increased community ANPP (Deutsch, Bork, & Willms, 2010a; Wang et al., 2011), but some studies found the opposite (Kelemen, Török, Valkó, Miglécz, & Tóthmérész, 2013). Moreover, litter accumulation can alter biogeochemical nutrient cycles through its decomposition (Moretto, Distel, & Didoné, 2001; Wang, Xu, et al., 2017), as well as soil bacterial components and diversity (Hossain, Okubo, & Sugiyama, 2010; Zeng, An, & Liu, 2017). Those alterations, in turn, modify plant–soil interactions (Brearley, Press, & Scholes, 2003). Unfortunately, limited information is available for understanding the role of those changes in the context of litter accumulation.

Litter accumulation can redistribute light, heat, and water, all of which have complex impacts on abiotic environment (Facelli & Pickett, 1991a, 1991b; Jensen & Gutekunst, 2003). Litter acts as a mechanical barrier, intercepting light, and altering the spectral structure (Facelli & Pickett, 1991b; Jensen & Gutekunst, 2003). It also reduces soil temperature during the day by decreasing solar radiation absorption, but increases soil temperature at night through reducing heat loss (Facelli & Pickett, 1991a). Additionally, litter accumulation may delay the freezing of soil in winter and thawing in spring (Facelli & Pickett, 1991a). Decreased soil temperature also indirectly improves soil moisture (Deutsch et al., 2010a). Litter can directly increase soil moisture by reducing water evaporation (Deutsch, Bork, & Willms, 2010b). In addition, litter accumulation can increase snow capture and retention in winter and soil moisture in early spring (Naeth & Chanasyk, 1995; Wikeem, Newman, & Ryswyk, 1989), but the interception effect of litter reduces infiltration by rainfall (Naeth, Bailey, Chanasyk, & Pluth, 1991). Therefore, understanding the modified microenvironment is crucial to elucidate the role of litter accumulation in regulating plant communities in fenced grasslands. Previous studies in fenced grasslands failed to continuously measure abiotic factors (Deutsch et al., 2010a; Facelli & Pickett, 1991a; Wang et al., 2011), and little is known about the effects of continuous changes in abiotic factors at different stages of the growing season.

The grasslands in Inner Mongolia are typical of grasslands in northern China. Bunch grass and rhizome grass grasslands are the most common types and are widely distributed in this region (Kang et al., 2007). However, because overgrazing has caused severe grassland degradation over the past decades, (Wang, Deng, Song, Li, & Chen, 2017), long‐term fencing has been widely implemented. Community structure and function have been significantly altered in some region due to excess litter accumulation, particularly in these bunch grass and rhizome grass grasslands.

The purpose of this study was to elucidate the role of excess litter accumulation in regulating light quantity, soil temperature, and soil moisture, and driving community successional changes in two fenced grasslands in Inner Mongolia. To this end, we performed a three‐year field experiment in two contrasting fenced grasslands. We hypothesized that excess litter accumulation could directly affect light, heat, and water regimes, with subsequent effects on community height, ANPP, and the relative dominance of different functional groups in fenced grasslands.

## 2 MATERIALS AND METHODS

### 2.1 Study area

The study area was located in Xilin Gol League, Inner Mongolia. Grasslands are the typical vegetation, most of which have been fenced for a long time. *Stipa grandis* (bunch grass) and *Leymus chinensis* (rhizome grass) grasslands were selected as experimental communities. The *S. grandis* grassland was located at the Inner Mongolia Grassland Ecosystem Research Station of the Chinese Academy of Sciences (IMGERS, 43°33′37″N, 116°40′12″E, 1,244 m). The *L. chinensis* grassland was located at the Grassland Ecological Research Station of Inner Mongolia University (GERSIMU, 44°09′44″N, 116°29′08″E, 1,102 m). The two study sites have extensive and long‐term fenced grasslands with typical and homogeneous vegetation.

The study area belongs to a temperate continental monsoon climate (cold and dry in winter and hot and wet in summer). The mean annual temperature of the *S. grandis* grassland is 0.3°C, with mean monthly temperature ranging from −21.6°C in January to 19.0°C in July. The mean annual precipitation is 351.0 mm, and 80% of the precipitation usually occurs from May to August. The mean annual temperature of the *L. chinensis* grassland is 0.1°C. The temperature of the coldest and hottest month is −19.0°C in January and 21.4°C in July; the mean annual precipitation is 300.3 mm. The soil type of the two study sites is a chestnut soil (Chinese Soil Taxonomic Classification), and the clay content is higher in the *L. chinensis* grassland than in the *S. grandis* grassland. The growing season in late April and lasts to mid‐September.

In the *S. grandis* grassland, the common species include *L. chinensis*, *Agropyron cristatum*, *Cleistogenes squarrosa*, *Achnatherum sibiricum*, *Carex duriuscula*, * Allium condensatum*, and *Allium tenuissimum*. In the *L. chinensis* grassland, the common species include *C. squarrosa*, *Stipa krylovii*, *C. duriuscula*, *Lappula myosotis*, and *A. tenuissimum*. The number of species is lower in the *L. chinensis* grassland than in the *S. grandis* grassland.

Based on climate data at the two study sites, air temperature was higher in 2017 than in 2015 and 2016, and the precipitation was higher in 2015 and 2016 than in 2017 (Table 1). We also observed that plants suffered from drought stress in the early growing season of 2017.

**Table 1:** Climate data over the growing season at the two study sites during 2015–2017

Study sites	Year	Growing season					Average	Sum
		May	Jun.	Jul.	Aug.	Sept.		
Temperature (°C)								
IMGERS	2015	10.9	15.5	19.3	17.7	12.0	15.1	—
	2016	12.2	16.2	21.1	20.2	12.0	16.3	—
	2017	13.6	17.5	22.2	17.9	13.0	16.8	—
GERSIMU	2015	10.8	15.6	20.0	17.3	13.3	15.4	—
	2016	14.2	16.1	21.8	20.3	11.5	16.8	—
	2017	13.2	18.4	22.5	17.9	15.4	17.5	—
Precipitation (mm)								
IMGERS	2015	24.7	85.8	51.8	41.2	52.9	—	256.4
	2016	30.2	35.2	68.0	40.2	60.9	—	234.5
	2017	9.8	16.9	69.1	82.5	24.8	—	203.1
GERSIMU	2015	33.2	75.6	74.2	16.0	5.3	—	204.3
	2016	8.4	41.7	71.2	26.6	52.2	—	200.1
	2017	6.1	25.7	53.1	42.7	11.9	—	139.4

### 2.2 Experimental design

Fenced enclosures (1,000 m × 800 m) were established in the two grasslands for 5 years, from 2009 to 2014, and excess litter was left to accumulate on the soil surface. Before 2009, the *S. grandis* grassland had experienced light degradation but the *L. chinensis* grassland was heavily degraded.

To elucidate the role of excess litter accumulation in regulating abiotic environment and plant communities, we conducted a 3‐year field experiment with a random complete block design. Three replicate blocks (22 m × 50 m) were established within the *S. grandis* and *L. chinensis* grasslands on flat, open terrain with homogeneous vegetation. For each grassland, the distance between two blocks was 200 m to ensure the independence of blocks and the length of each block reached up to 50 m to decrease vegetation heterogeneity. Each block was divided into half (10 m × 50 m) with a 2‐m buffer in the middle. Each half was randomly assigned either to the treatment where the litter was completely removed or to the control where litter was left to accumulate. In this study, litter included the dead, aboveground, intact, or partially decomposed plant material (mainly leaves and stems of plants) on the soil surface. The litter in the control plots was completely retained during 2015–2017. At the end of each growing season during 2014–2016, the litter in the litter removal treatment was cut near the soil surface level with a mower and removed with a rake (Wang et al., 2011). Monitoring environmental factors and vegetation began in May 2015.

### 2.3 Measurements of soil temperature and soil moisture

Soil temperature and soil moisture were measured in situ with ECH_2_O 5TE sensors (METER Company). For one of the three blocks in each grassland, a flat and open terrain was selected and ECH_2_O 5TE sensors were inserted at depths of 2.5 cm and 12.5 cm, representing the upper and deeper soil layers affected by litter accumulation. After inserting the sensors, soil was backfilled and the surface was smoothed to prevent pooling during rainfall. Soil temperature and soil moisture were recorded at 10‐min intervals from 1 May 2015 to 30 September 2017.

### 2.4 Measurements of light quantity

Light quantity was measured with an array illuminometer (ZL2016 2 1344510.1) designed by us. This design can reduce the heterogeneity in light estimates due to litter accumulation. The array illuminometer was composed of five light quantity sensors arranged in a row at 10‐cm intervals. An adjustable shelf allowed us to measure light quantity at different heights. First, the array illuminometer was placed at a random location within the control plot and the light quantity at the soil surface was measured. Then, the array illuminometer was elevated at 5‐cm intervals to measure the light quantity at different heights. Finally, the light quantity that was unshaded by litter was measured (i.e., full light quantity). Light quantity at a given height was recorded with five light quantity sensors (five replicates per height) when the reading of the array illuminometer was stable. Light quantity was measured between 14:00 and 15:00 with cloudless weather and at 6‐day intervals from mid‐April to mid‐May during 2015–2017.

### 2.5 Plant community sampling

Plant community characteristics were sampled using three quadrats (1 m × 1 m) randomly placed within each block half. For each quadrat, we recorded species composition and measured plant height. All the litter in the quadrat of the control was harvested. The aboveground biomass of each living plant was also harvested. The litter and aboveground biomass were oven‐dried at 65°C for 48 hr and weighted. Plant communities were sampled on the first day of each month (June to September) during 2015–2017.

### 2.6 Data analyses

Because the litter in the litter removal treatment was removed during 2015–2017, litter accumulation was only in the control, indicated by the mean dry litter biomass in the control plots in June of 2015–2017.

Percent light interception indicated the light regime in the experiment. Because there were no litter and plants in the litter removal treatment in the early growing season, light quantity at different heights was indicated by full light quantity. Percent light interception in the control plots was calculated with the following equation (Deutsch et al., 2010b).

$$\text{Percent}\;\text{light}\;\text{interception}=(1-\frac{{\text{Li}}_{C}}{{\text{Li}}_{E}})\times 100\%,$$

where Li_C_ is the light quantity at different heights in the control. Li_E_ is the light quantity at the same height in the litter removal treatment, which was full light.

The effects of litter accumulation on soil temperature and soil moisture were indicated by the difference in soil temperature and soil moisture between the litter removal treatment and control (Yan et al., 2018). The larger the difference, the greater the effects of litter accumulation. We used daily mean soil temperature and soil moisture at the same depth to calculate the difference (1 May to 30 September of 2015–2017). The two equations were as follows:

$$\text{Soil}\;\text{temperature}\;\text{difference}={\text{ST}}_{C}-{\text{ST}}_{E}$$

$$\text{Soil}\;\text{moisture}\;\text{difference}={\text{SM}}_{C}-{\text{SM}}_{E}$$

where ST_C_ is the daily mean of soil temperature in the control, and ST_E_ is the daily mean of soil temperature in the litter removal treatment at the same depth. Similarly, SM_C_ is the daily mean of soil moisture in the control and SM_E_ is the daily mean of soil moisture in the litter removal treatment at the same depth.

Mean community height and ANPP were calculated across the nine quadrats per litter removal treatment and per control. We classified all species into bunch grasses, rhizome grasses, perennial forbs, and annual plants. Bunch grasses and rhizome grasses were dominant in our study. Bunch grasses included *S. grandis*, * C. squarrosa*, and *A. cristatu*. Rhizome grasses included *L. chinensis* and *C. duriuscula*. The relative dominance of different plant functional groups was indicated by the corresponding monthly mean of relative dry biomass in the nine quadrats, as follows:

$$\text{Relative}\;\text{dominance}=\frac{B_{i}}{B}\times 100\%$$

where *B_i_* is the dry biomass of a plant functional group in a given quadrat, and *B* is the total dry biomass of the same quadrat.

One‐way analysis of variance with a post hoc Tukey test was used to test for differences in litter accumulation and percent light interception at each height during 2015–2017. A general linear mixed effects model was used to test the effects of litter accumulation on community height and ANPP with treatment as a fixed effect and block as a random effect. The linear recursive analysis was selected to quantify relationships between the relative dominance of two plant functional groups and both soil temperature and soil moisture. All statistical analyses were performed using SPSS 21.0 (IBM).

## 3 RESULTS

### 3.1 Litter accumulation characteristics

**Figure 1:**
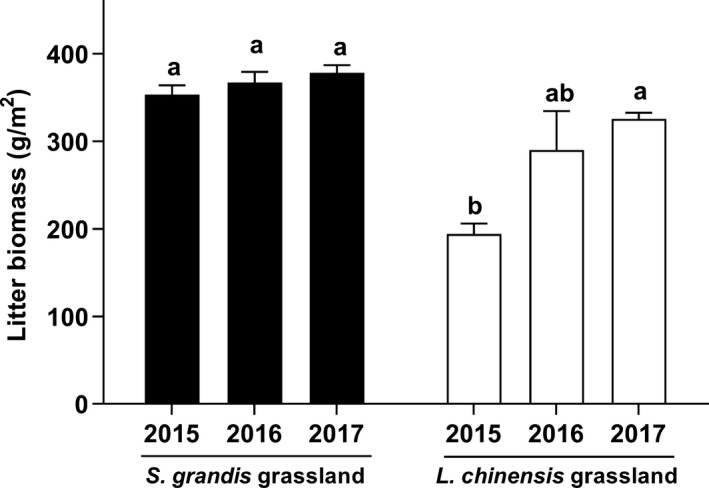
Dynamics of litter accumulation in the control plots of two grasslands. Data are means + 1 *SE* and *N* = 9; different lowercases indicate there are significant differences in litter biomass during 2015–2017 at 0.05 level

Litter biomass increased rapidly and significantly in the control plots of the *L. chinensis* grassland (Figure 1; *p* < .05) during 2015–2017, but did not change in the *S. grandis* grassland (Figure 1; *p* > .05).

### 3.2 Light quantity characteristics

Litter accumulation reduced the light quantity in the control plots of both grasslands (Table 2). Percent light interception at the soil surface averaged 97.2% in the control plots of the *S. grandis* grassland, higher than the average of 87.3% in the control plots of *L. chinensis* grassland (Table 2). Overall, percent light interception declined with community height in the control plots of both grasslands. However, interception declined rapidly in the *S. grandis* grassland but slowly in the *L. chinensis* grassland (Table 2). Due to litter accumulation, percent light interception significantly increased in the *L. chinensis* grassland between 2015 and 2017 (Table 2; *p* < .05).

**Table 2:** Percent light interception at different heights in the control plots of two grasslands

Community types	Year	Height (cm)					
		0	5	10	15	20	25
*S. grandis*	2015	98.6 ± 0.6^a^	93.9 ± 4.2^a^	68.1 ± 4.4^a^	58.2 ± 3.8^a^	21.7 ± 6.5^a^	8.3 ± 4.4^a^
	2016	95.8 ± 1.5^a^	81.8 ± 2.4^a^	68.5 ± 4.1^a^	45.7 ± 5.2^b^	22.4 ± 6.4^a^	9.1 ± 4.0^a^
	2017	97.1 ± 0.3^a^	76.6 ± 3.9^a^	62.2 ± 4.5^a^	41.3 ± 6.2^b^	23.8 ± 2.2^a^	8.7 ± 2.9^a^
*L. chinensis*	2015	83.7 ± 3.9^b^	58.8 ± 3.7^b^	51.1 ± 8.1^b^	36.0 ± 4.6^b^	19.3 ± 3.1^b^	17.7 ± 2.7^b^
	2016	90.1 ± 1.8^a^	72.1 ± 2.4^a^	71.9 ± 3.2^a^	47.5 ± 9.8^b^	48.8 ± 2.5^a^	30.8 ± 7.0^a^
	2017	88.1 ± 1.6^a^	77.1 ± 3.1^a^	71.8 ± 5.2^a^	60.9 ± 7.9^a^	40 ± 7.2^a^	18.9 ± 3.8^b^

Note

Data are means ± 1 *SE* and *N* = 25; different lowercases in the same community indicate significant differences during 2015–2017 at 0.05 level.

### 3.3 Soil temperature characteristics

**Figure 2:**
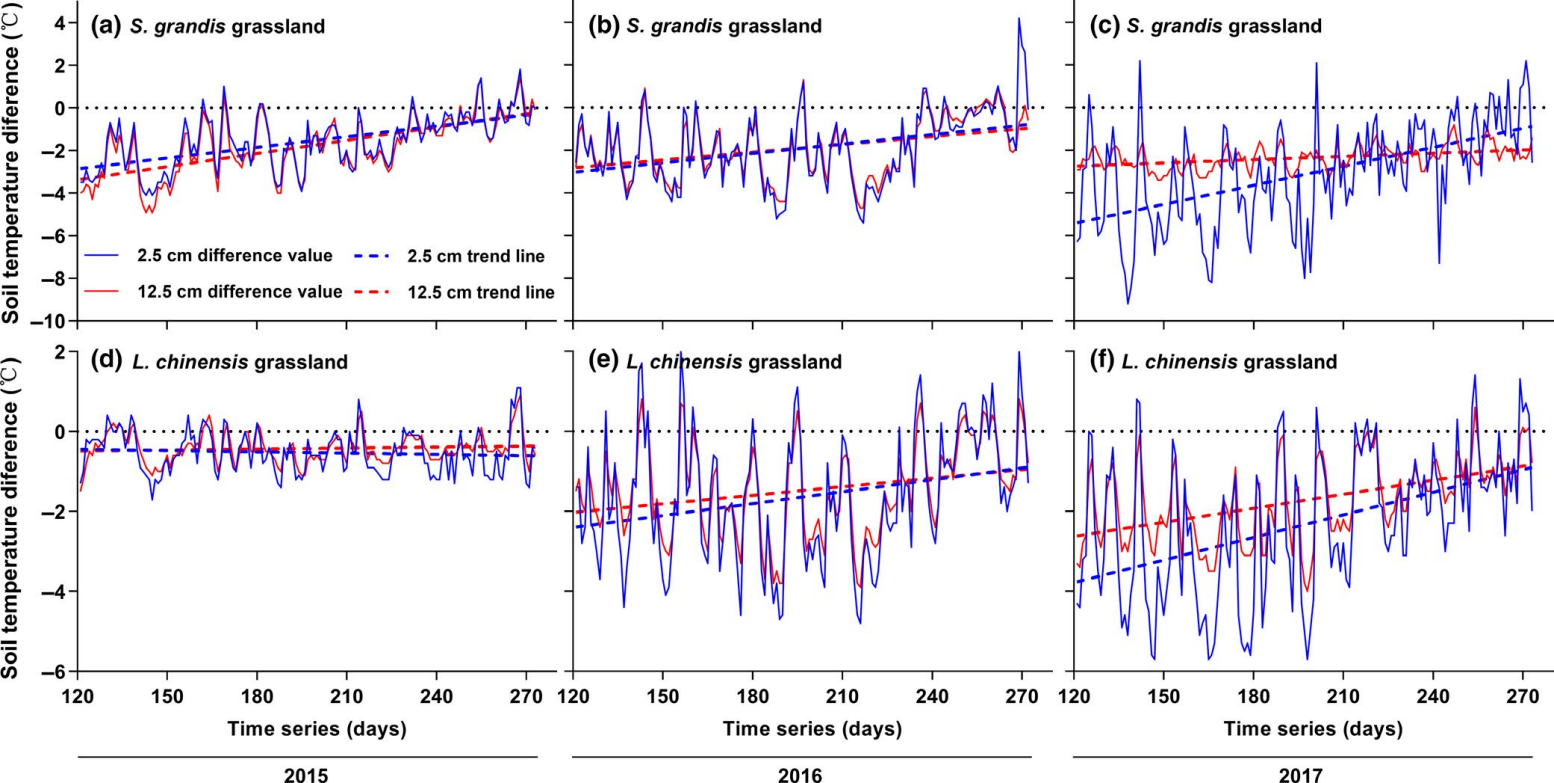
Dynamics of soil temperature difference and trend of soil temperature difference in two grasslands. Time series are the sequence of the Gregorian calendar; lines are soil temperature difference between litter removal treatment and control; dotted lines are trend lines of soil temperature difference during the growing season

Litter accumulation decreased soil temperature during the growing season at both 2.5 cm and 12.5 cm depths in the control plots of both grasslands (Figure 2). The difference in soil temperature between the litter removal treatment and control was greater at 2.5 cm than at 12.5 cm (Figure 2). The difference in soil temperature gradually decreased over time (Figure 2, the original data were supplemented in Figure S1). Specifically, the soil temperature difference was greater earlier in the growing season than later (Figure 2). This difference indicated that the regulating effect of litter accumulation on soil temperature fluctuated, and importantly, gradually weakened over the growing season. In addition, compared with 2015, soil temperature differences in 2016 and 2017 were higher in the *L. chinensis* grassland (Figure 2).

### 3.4 Soil moisture characteristics

**Figure 3:**
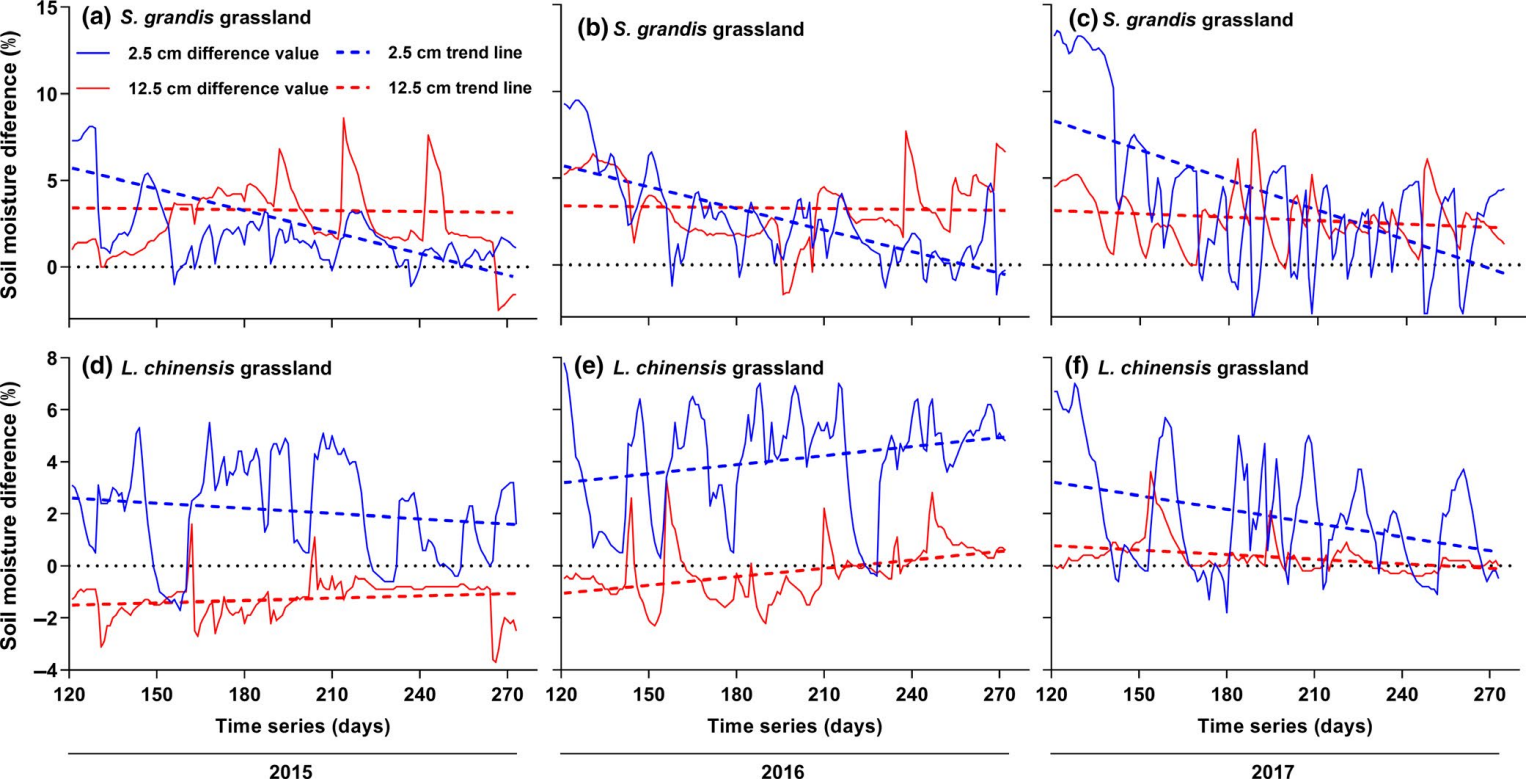
Dynamics of soil moisture difference and trend of soil moisture difference in two grasslands. Time series are the sequence of the Gregorian calendar; lines are soil moisture difference between litter removal treatment and control; dotted lines are trend lines of soil moisture difference during the growing season

Unlike soil temperature, litter accumulation increased soil moisture at both 2.5 cm and 12.5 cm depths in the control plots of both grasslands during the growing season (Figure 3, the original data were supplemented in Figure S2). The variation in soil moisture at 2.5 cm was greater than at 12.5 cm (Figure 3). The soil moisture difference gradually decreased over the growing season, particularly at 2.5 cm (Figure 3).

### 3.5 Plant community characteristics

**Figure 4:**
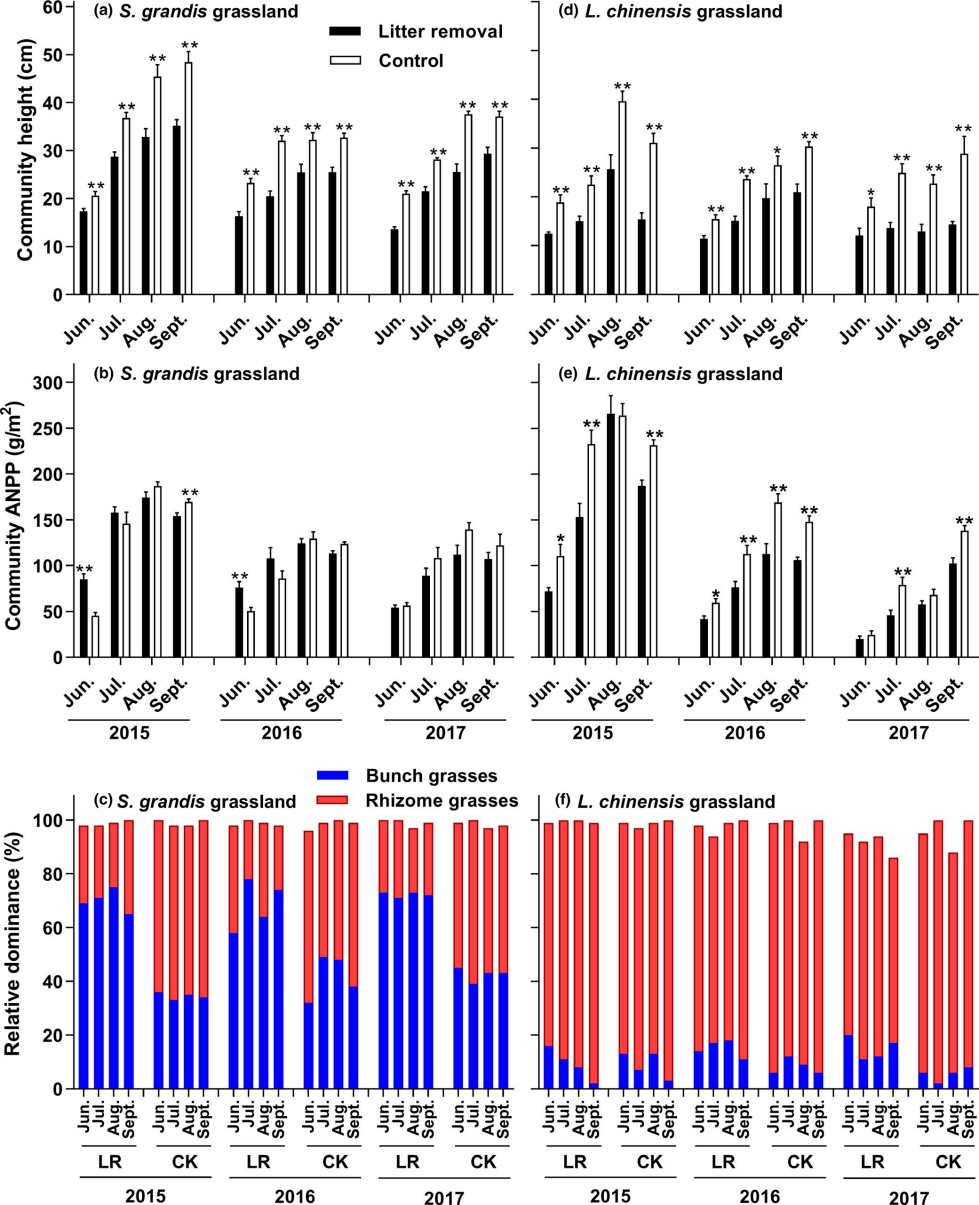
Dynamics of plant community characteristics in two grasslands. LR: litter removal, CK: control; data are means + 1 *SE* and *N* = 9; * and ** indicate that community height and ANPP have significant differences between the two treatments at 0.05 level and 0.01 level

Litter accumulation significantly increased community height in the control plots of both grasslands during the growing season (Figure 4a,d; *p* < .01). Specifically, in August of 2015 and 2016, community height was 27.2%–38.4% and 33.8%–54.1% greater in the control than the litter removal treatment of the *S. grandis* and *L. chinensis* grasslands (Figure 4a,d). In 2017, community height increased by up to 46.5% in the *S. grandis* grassland and 76.0% in the *L. chinensis* grassland (Figure 4a,d). In other words, litter accumulation altered community vertical structure.

Litter accumulation generally increased community ANPP in the control compared with the litter removal treatment (Figure 4b,e). In August of 2015 and 2016, community ANPP was 4.0%–7.3% greater in the control plots of the *S. grandis* grassland in the middle and late growing season, but was lower in the early growing season (Figure 4b; *p* < .05). However, litter accumulation always increased community ANPP (−0.8% to 49.6% in August) in the control of the *L. chinensis* grassland in these years (Figure 4e; *p* < .01). Furthermore, in 2017, a relatively dry year, the effect of litter was more evident in increasing community ANPP (Figure 4b,e), particularly in the *S. grandis* grassland (24.4% in August).

Litter accumulation altered the relative dominance of the two plant functional groups in both grasslands (Figure 4c,f). In the control plots of the *S. grandis* grassland, litter accumulation significantly increased the relative dominance of rhizome grasses but decreased that of bunch grasses (Figure 4c). However, litter accumulation had no effect on the relative dominance of rhizome grasses but slightly decreased that of bunch grasses in the control plots of the *L. chinensis* grassland (Figure 4f).

**Figure 5:**
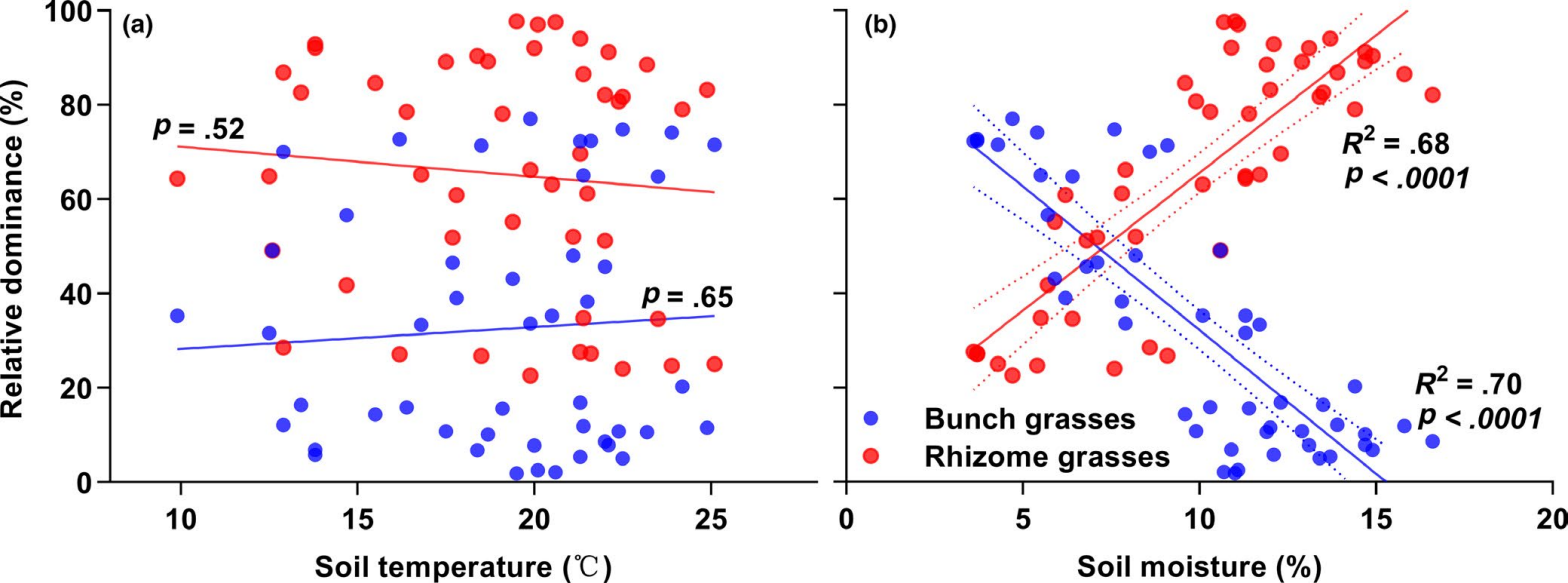
Relationships between the relative dominance of two plant functional groups and soil temperature and soil moisture. Soil temperature and soil moisture were monthly means between the depths of 2.5 cm and 12.5 cm during the growing season; dotted lines are the 95% confidence intervals of the fitting lines

Across both grasslands, the relative dominance of rhizome grasses and bunch grasses had no significant relationship with soil temperature (Figure 5a; *p* > .05). However, greater soil moisture led to an increase in the relative dominance of rhizome grasses and an decrease in bunch grasses (Figure 5b; *p* < .0001). A total of 68% of the variation in rhizome grasses and 70% of the variation in bunch grasses were explained by soil moisture (Figure 5a,b). Thus, the increase in soil moisture due to litter accumulation could be a driving force to shift the relative dominance of two plant functional groups in fenced grasslands.

## 4 DISCUSSION

Overall, litter accumulation strongly altered light quantity, soil temperature and moisture, and drove community successional changes in two fenced grasslands in Inner Mongolia. The effects of litter accumulation on soil temperature and moisture varied but gradually weakened over the growing season. The increase in soil moisture facilitated the relative dominance of rhizome grasses but suppressed that of bunch grasses. Consequently, the increased soil moisture associated with litter accumulation could potentially shift grasslands currently dominated by the bunch grasses to grasslands dominated by the rhizome grasses.

### 4.1 Effects of litter accumulation on soil temperature and soil moisture

In this study, litter accumulation decreased soil temperature but increased soil moisture, which is consistent with previous studies (Deutsch et al., 2010a, 2010b; Facelli & Pickett, 1991a). Early in the growing season, the shading effect of litter accumulation decreased soil temperature by preventing the absorption of solar radiation but increased soil moisture by inhibiting evaporation (Facelli & Pickett, 1991a). Solar radiation increased by the middle and late growing season, soil temperature increased, and the soil temperature difference between the control and litter removal treatment gradually diminished, especially later. The combined growth of plants and the rise in air temperature increased the loss of soil moisture due to evaporation and transpiration (Lauenroth & Bradford, 2006). However, because of concentrated rainfall in this region, water was continuously input into the soil in the middle and late growing season. Further, the accumulated litter also trapped and retained more snow in winter, which could increase soil moisture in spring (Naeth & Chanasyk, 1995). The balance of these factors resulted in the decrease in soil moisture in the litter removal treatment in the late growing season.

The effects of litter accumulation on soil temperature and soil moisture were strongest in the early growing season and had a negative effect on plant growth. Lower soil temperature can delay seed germination, decrease the growth rate of plants (Deutsch et al., 2010b), and even reduce community ANPP in the control plots of both grasslands (Figure 4b,e). But because of the increase in plant growth over the growing season, the effect of litter accumulation on these plants was slowly reduced. Later in the year, the soil surface covered by litter maintained a warm and stable environment, extending the growing season (Facelli & Pickett, 1991a; Watt, 1970). Similarly, relatively higher soil moisture allowed plants to resist drought stress in the middle and late growing season, thereby increasing community ANPP (Figure 4b,e). Over longer time scales, the abiotic environment tended to become shaded and moist. Species likely have different adaptations to these changes, which altered the original community's reproduction, interspecific competition, composition, and structure. In particular, the number of moisture‐tolerant species increased and the number of drought‐tolerant species decreased.

The soil moisture difference between the litter removal treatment and control was greater in 2017, a relatively dry year, than in 2015 and 2016 (Figure 3). This finding indicates that litter accumulation might have a greater regulating effect on soil moisture in dry years. It also highlights that litter could play an important role in regulating water circulation and increasing soil water availability in arid and semiarid grassland ecosystems in the future as climate changes. Furthermore, as litter continued to accumulate, the effects of litter accumulation on soil temperature and soil moisture were stronger in 2016 and 2017 than in 2015 in the control plots of the *L. chinensis* grassland (Figures 2 and 3). This result indicates that some threshold of litter accumulation may determine the degree of its regulating effects (Deutsch et al., 2010b; Loydi, Eckstein, Otte, & Donath, 2013).

### 4.2 Effects of litter accumulation on light quantity

Light quantity was reduced where the litter was not removed (Table 2), which agrees with previous studies (Facelli & Pickett, 1991b; Jensen & Gutekunst, 2003; Weltzin et al., 2005). In our study, percent light interception decreased rapidly in the control plots of the *S. grandis* grassland but decreased slowly in the control plots of the *L. chinensis* grassland (Table 2). These contrasting changes might be due to differences in the litter in the two grasslands. Most of the litter lay flattened on the soil surface in the *S. grandis* grassland but remained standing for a long time in the *L. chinensis* grassland. In the presence of wind and snow, litter was also more likely to concentrate on the soil surface in the *S. grandis* grassland than the *L. chinensis* grassland. Further, tall plants are often better competitors for light than dwarf plants, especially when litter accumulation could intensify this competition (Letts et al., 2015). In addition, reduced light quantity delayed the increase in soil temperature, especially in the early growing season.

### 4.3 Effects of litter accumulation on plant community

In our study, we found that the increase in soil moisture led to rhizome grasses expanding rapidly and bunch grasses declining, particularly in the *S. grandis* grassland (Figure 4c,f). These changes might alter interspecific competition between rhizome and bunch grasses, which was due to the fact that different plant functional groups respond differentially to water availability (Figure 5). Rhizome grasses are often moisture‐tolerant species, and bunch grasses are usually drought‐tolerant species (Chen, Bai, Zhang, & Han, 2005). Thus, the moister microenvironment where litter accumulated benefited the rhizome grasses more than the bunch grasses. With the expansion of rhizome grasses and decline of bunch grasses, grassland resources could be improved because livestock in this region prefer to consume rhizome grasses.

In addition, litter accumulation can negatively affect the sexual reproduction of plants (Deutsch et al., 2010b), potentially impacting the population growth of species with sexual reproduction, such as *S. grandis* and *C. squarrosa*, whereas asexually reproducing species such as *L. chinensis* and *C. duriuscula* might be less affected. Moreover, the lack of external stimuli, such as grazing and mowing, might also inhibit the tillering of bunch grasses, further limiting their growth and reproduction.

Our findings suggest that litter accumulation potentially drove community successional changes in bunch grass grasslands and that long‐term fencing facilitates this shift. Succession usually occurs over long‐time scales in arid and semiarid grasslands, but litter accumulation might act as a medium to indirectly alter interspecific competition and accelerate this process.

Litter accumulation significantly increased community height (Figure 4a,d). This change may be the outcome of competition for water and/or light. The increase in community height altered the vertical structure of community and could have an asymmetrical effect on the growth of different species in these fenced grasslands. Litter accumulation could stimulate the growth of all plants, or only promote height growth of tall plants while inhibiting the growth of short plants because of the mechanical barrier of litter and the shading effect of tall plants.

Litter accumulation could promote community ANPP (Figure 4b,e), which agrees with some previous studies (Weltzin et al., 2005; Willms, McGinn, & Dormaar, 1993). This increase in ANPP was likely due to the increase in soil moisture (Deutsch et al., 2010b; Wang et al., 2011). Interestingly, the community ANPP of the *S. grandis* grassland decreased in the early growing season. Compared with the *L. chinensis* grassland, the litter was denser (Figure 1) and more concentrated on the soil surface in the *S. grandis* grassland. These differences could delay seed germination and decrease plant growth in the *S. grandis* grassland in the early growing season. The increase in community ANPP was higher in the *L. chinensis* grassland than the *S. grandis* grassland (Figure 4b,e). This result was consistent with rapid litter accumulation in the *L. chinensis* grassland during 2015–2017 (Figure 1) and indicates that rhizome grass grasslands may be more suitable for litter accumulation than bunch grass grasslands. In a relatively dry year (2017), we observed that litter accumulation promoted community ANPP, supporting previous findings (Deutsch et al., 2010b).

Note that the litter removal was achieved via mowing at the end of the growing season, and the litter in the control plots was completely retained in this study. Ideally, the litter in these two treatments should be removed at the same time and the corresponding litter was re‐applied to the control plots of two grasslands. However, this manipulation could strongly destroy the natural structure of litter layer in the control plots, thereby altering the real light, heat, and water regimes. In this study, we used mowing to remove litter for the following reasons. First, our blocks (20 m × 50 m) were much larger than those (2 m × 6 m) used in previous studies (Wang et al., 2011). It was hardly possible to remove litter from such large blocks without external interference. In order to uniformly remove litter, mowing might be the most feasible method. Secondly, this method has been widely applied in similar studies in Inner Mongolia (DJ Hou, personal observation). To minimize the effects of mowing, litter removal is commonly conducted at the end of growing seasons when all plants are dormant (Deutsch et al., 2010b; Wang et al., 2011). However, it should be noted that litter removal by mowing has some limitations. First, mowing can affect plants due to the presence of mechanical disturbances, such as increasing soil compaction and trampling plants. Secondly, the mechanical disturbance due to mowing should be applied in the control plots, but this manipulation could alter the natural structure of litter layer, leading to the absence of real control. In the future, similar studies about litter removal by mowing should consider the effects of mechanical disturbances as much as possible, and the effects of litter removal and mechanical disturbances should be dissected.

In summary, our three‐year field experiment provides insights into the role of litter accumulation in regulating abiotic factors and plant communities in fenced grasslands. Our findings will advance our understanding of community succession in the context of litter accumulation. Litter accumulation regulated light quantity, soil temperature, and soil moisture, increased community height and ANPP, and shifted the relative dominance of different plant functional groups. The vegetation and ecosystem services of degraded grasslands were improved after appropriate fencing. In addition, forage palatability was increased because of the increase in rhizome grasses.

## CONFLICT OF INTEREST

No conflict of interest.

## AUTHOR CONTRIBUTION

Ke Guo, Changcheng Liu, and Dongjie Hou conceived and designed this experiment. Dongjie Hou and Xianguo Qiao performed the field experiment and processed the data. Dongjie Hou and Weiming He analyzed the data and wrote the manuscript.

## Data Availability

The data supporting the results are archived on Dryad (https://doi.org/10.5061/dryad.6r99k25).
