# Supplementary figures and images for: Litter accumulation alters the abiotic environment and drives community successional changes in two fenced grasslands in Inner Mongolia
Source: Ecol Evol. 2019 Jul 23;9(16):9214–24. doi: 10.1002/ece3.5469 (PMC6706195; doi:10.1002/ece3.5469)

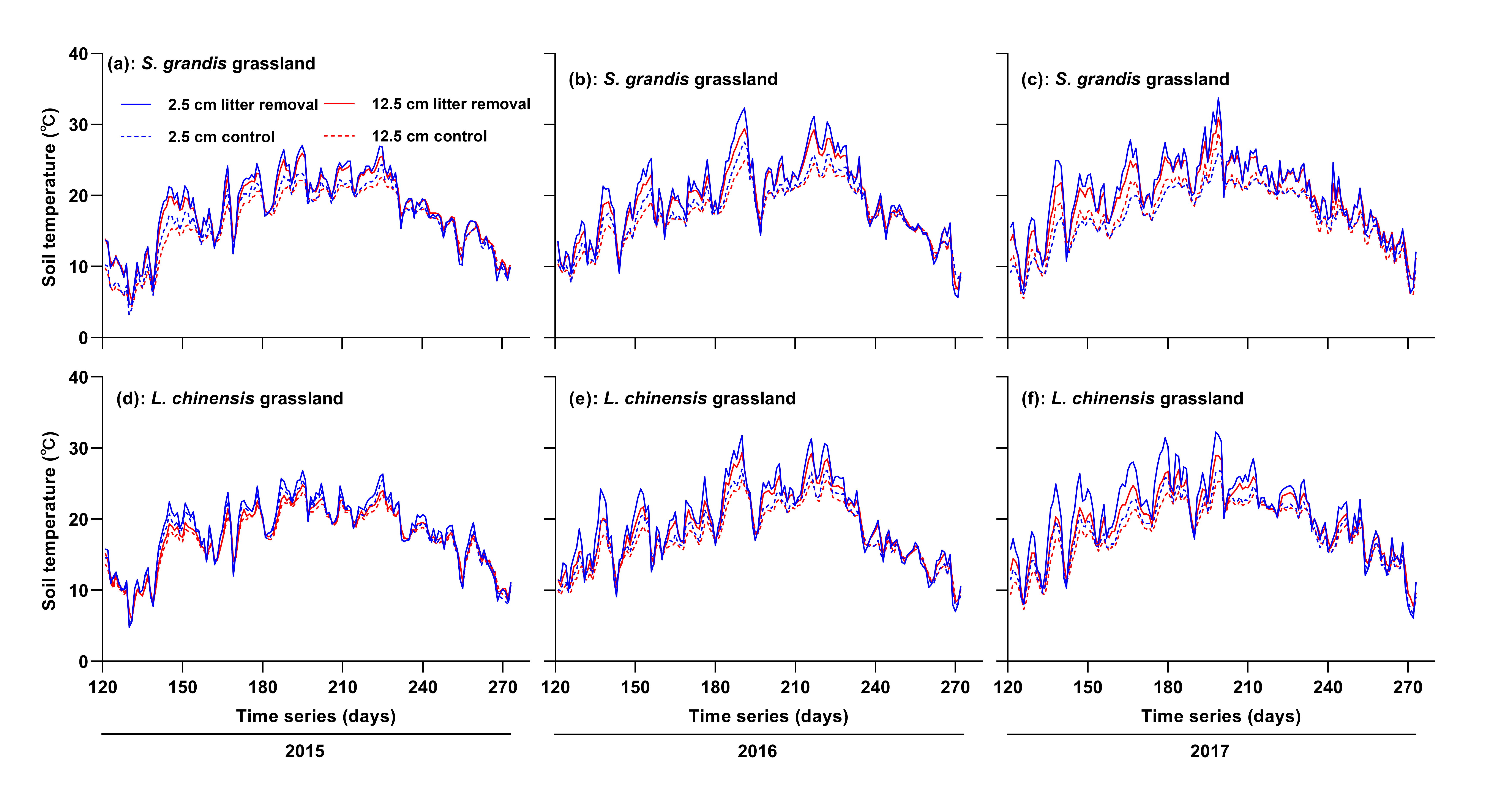

Supplement: Supplementary file 1 [file ECE3-9-9214-s001.tif]

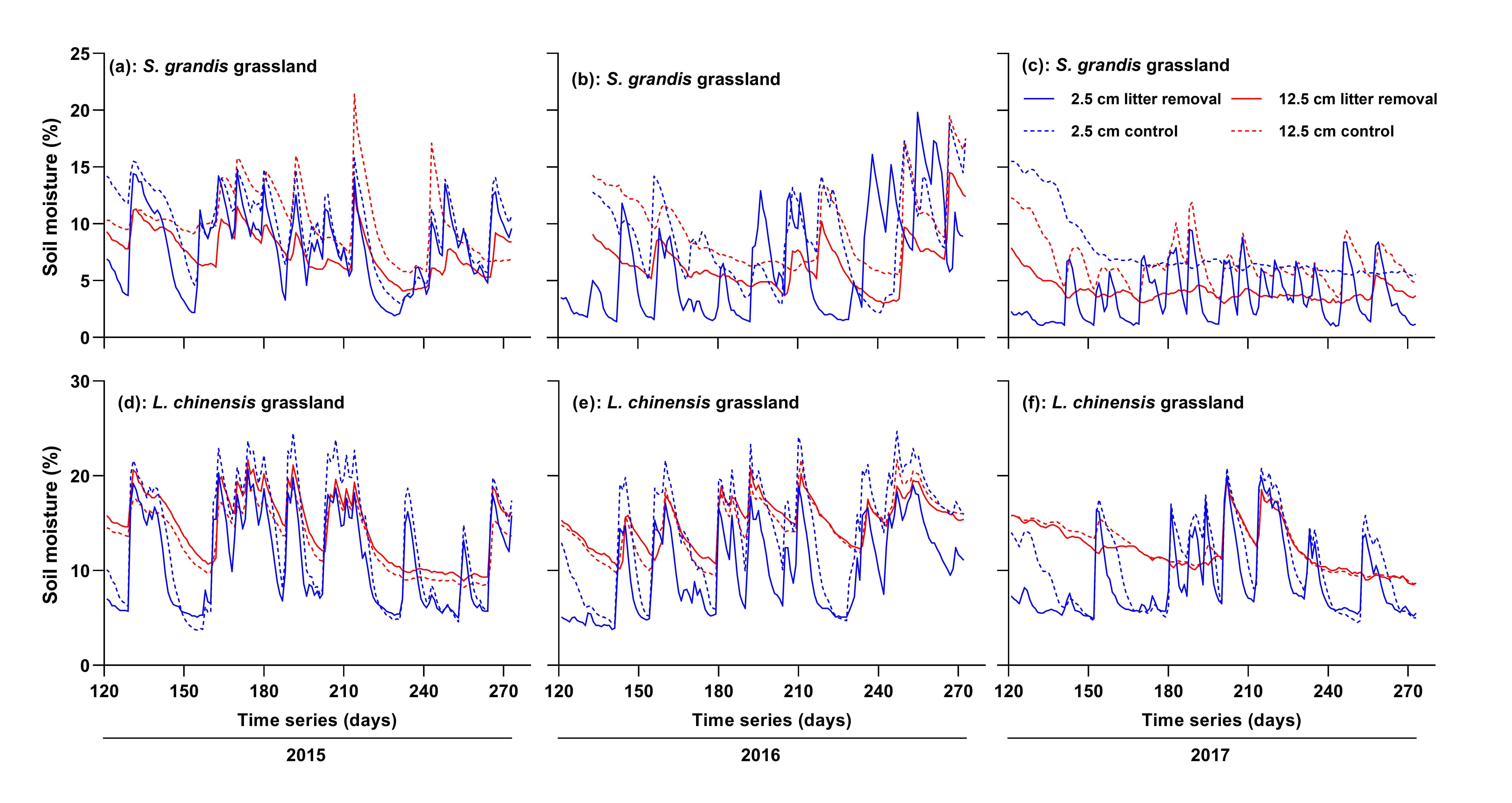

Supplement: Supplementary file 2 [file ECE3-9-9214-s002.tif]
